# Current status of international experiences in general surgery residency programs in the United States

**DOI:** 10.1186/s40064-016-2270-x

**Published:** 2016-05-11

**Authors:** Filipe A. Sobral, Alexis N. Bowder, Lynette Smith, Advaitaa Ravipati, Melissa K. Suh, Chandrakanth Are

**Affiliations:** Mount Sinai Doctors, Long Island, 325 Park Ave., Huntington, NY 11743 USA; College of Medicine, University of Nebraska Medical Center, 983280 Nebraska Medical Center, Omaha, NE 68198 USA; College of Public Health, University of Nebraska Medical Center, 983280 Nebraska Medical Center, Omaha, NE 68198 USA; University of South Florida, 4202 E. Fowler Ave., Tampa, FL 33620 USA; Department of Surgery, University of Nebraska Medical Center, 983280 Nebraska Medical Center, Omaha, NE 68198 USA

**Keywords:** Education, Global surgery, Training

## Abstract

**Background:**

The aim of this study is to identify, quantify, and characterize the international experiences available for general surgery residents in the general surgery residency programs in the United States (US).

**Methods:**

The Fellowship and Residency Electronic Interactive Database (FREIDA) database was used to identify all the general surgery residency programs in the US. Each institution was contacted by both e-mail and telephone. Respondents were asked if an international experience was available for residents within their program and, if available, details of the experience were obtained.

**Results:**

A total of 253 general surgery residency programs were identified using the FREIDA database. Eighty-six (34 %) programs were noted to offer an international experience for their residents. A majority are incorporated into the PGY 3 and PGY 4 level of training with a duration of 1–4 weeks. Common locations are evenly distributed among the Americas and Africa and are usually funded through a combination of resident and department funding.

**Conclusions:**

US resident interest in global health is growing along with an increasing demand for surgical care worldwide. This is one of the first studies to identify, quantify, and characterize in detail the international experiences currently available to general surgery residents within the general surgery training programs in the US. These results can help general surgery residency applicants with an interest in global health and also pave the path for residency programs to develop future international experiences.

## Background

The number of deaths due to surgical conditions worldwide was estimated to be 16.9 million in 2010 and well exceeded the number of deaths due to HIV/AIDs, tuberculosis, and malaria combined (Shrime et al. 2015; Lozano et al. 2012). The Lancet Commission on Global Surgery estimates that five billion people worldwide lack access to safe and affordable surgical and anesthetic care with the greatest disparity existing in low and middle-income settings (Meara and Greenberg 2015; Lett 2003). Organizations worldwide, including the World Health Organization and the Lancet Commission on Global Surgery, continue to recognize and advocate for an increased surgical presence in developing nations. They are working towards establishing goals for improved surgical care worldwide. Concurrently, there has been an increase in interest as well as recognition of the importance of international surgical experiences for general surgery residents in the United States (US).

Since 2011, when the Residency Review Committee and the American Board of Surgery approved international rotations to count toward graduation requirements, interest in pursuing international electives has increased (Mitchell et al. 2011; Accreditation Council for Graduate Medical Education 2015). Surveys of residents from individual institutions and national surveys have documented a significant interest among respondents in pursuing international surgical experiences (Powell et al. 2007, 2009; Klaristenfeld et al. 2008; Jayaraman et al. 2009). In addition, several programs have reported and documented their ongoing experiences with international surgery rotations along with their potential benefits (Klaristenfeld et al. 2008; Silverberg et al. 2007; Ozgediz et al. 2005, 2008).

Despite the growing need for surgical care worldwide and the rising interest expressed by general surgery residents and applicants in international rotations, there is currently no standard platform for international rotations in general surgery residency programs in the US (Barton et al. 2008). Predominately, residents are required to fund their own experiences and must use vacation time to pursue these activities (Powell et al. 2009). The information regarding international experiences during general surgical training has, so far, been largely extrapolated from web based surveys with relatively low response rates. These data, therefore, may not be an accurate representation of the actual opportunities available to US general surgery residents. There has been no formal study published with a detailed analysis of the existing global health experiences offered by US general surgery residency training programs (Powell et al. 2007, 2009). The aim of this study is to identify, quantify, and characterize in detail the international opportunities available for general surgery residents in the general surgery residency programs in the US.

## Methods

The Fellowship and Residency Electronic Interactive Database (FREIDA) database was used to preliminarily identify and stratify general surgery residency programs into three groups: programs offering international experiences, programs not offering international experiences, and programs where information regarding international experiences was unavailable (American Medical Association 2015). FREIDA is an online database of accredited graduate medical education programs maintained by the American Medical Association. It contains nearly 9000 graduate medical education programs recognized by the Accreditation Council for Graduate Medical Education.

After identification of general surgery residency programs, an email was sent to the program coordinators listed as the contact person from the FREIDA database from each institution and each program was contacted by telephone. Data was collected between December 2014 and April 2015. First, respondents were asked if an international experience existed within their program and was available to residents. An international experience was defined as a time where a current US general surgery resident travelled outside of the continental US to pursue a surgical experience. To ensure consistency in program response, this definition was communicated to the programs through the telephone conversation. Those who denied the existence of such opportunities or declined telephone interview were asked no further questions. The remaining programs were asked to provide further details of their international experiences including location, duration, post graduate year (PGY) of participation, funding source, amount of time the opportunity has been available to the residents, and resident participation to date. The experiences identified included formal elective rotations, global health tracks, mission trips, and experiences planned during vacation time. Programs with a global health track varied in the experiences provided ranging from a 2 month elective during the fourth year of residency to a 2 year commitment with dedicated time for pursuing a master’s in public health. These programs also had a dedicated curriculum throughout residency training for global health education, mentoring, and project planning. If a program contact person was unaware of specific details, that program not included in the subset analyses. If a program was unable to be reached on first attempt, a follow up phone call was made. If a program was unreachable on second attempt, a voicemail message was left explaining the general focus of the study and requesting a return phone call to a single telephone number.

## Results

A total of 253 US general surgery residency programs were identified using the FREIDA database. According to the FREIDA database 60 of these programs indicated offering international experiences, 108 did not indicate offering international experiences, and 85 were unknown (Table 1). Each of these programs was then contacted to confirm the information from the FREIDA database and further characterize the available international experiences.

**Table 1:** International experience availability

Rotation available	FREIDA	Survey results (telephone and e-mail)
Yes	60	86
No	108	153
Unknown	85	14
Total	253	253

Upon telephone or email confirmation, the number of programs found to have some form of international experience was 86 (34 %) while 153 (60 %) did not offer international experiences. There was no response by phone or email for 14 (6 %) programs with an overall response rate of 94 %. The information provided on the FREIDA database was found to be incorrect upon email or telephone confirmation in 24 of the programs surveyed. There were ten false negatives and 14 false positives identified out of the 239 respondents. False negatives were defined as programs listed as not having international experiences on the FREIDA database but found to have such opportunities upon telephone confirmation. False positives were those programs listed as having international experiences on the FREIDA website but were found to not provide this opportunity upon telephone or email interview. Unknown programs were those programs from which a telephone or email response was not obtained and no information regarding international work was available from the FREIDA database.

**Fig. 1:**
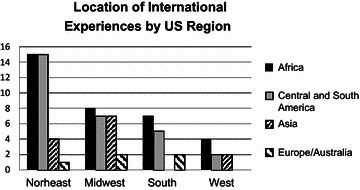
A *bar graph* demonstrating the most common international experience locations among each US region. Experiences in Africa and the Americas are the most common in programs across the US

The programs were then separated and evaluated based on geographic regions within the US. Geographic regions were determined according to the National Resident Matching Program assignments. We noted that the highest percentage of programs offering international experiences was in the West region (44 %) followed by the Midwest region (38 %), North East region (33 %), and South region (27 %) respectively (Table 2). A list of all the general surgery residency programs offering international experiences is summarized in Table 3. The locations for these experiences were also evaluated and we noted that Africa and the Americas were the most common destinations for international experiences (Fig. 1).

**Table 2:** Regional international experience availability

US region	Programs	International experience (%)
North East	86	28 (33)
Midwest	60	23 (38)
West	36	16 (44)
South	71	19 (27)
Total	253	86 (34)

**Table 3:** Programs with international experiences based on region

North East	Midwest	West	South
Berkshire Medical Center	Case Western Reserve University/University Hospitals Case Medical Center	College of Medicine Mayo Clinic (Arizona)	Carilion Clinic-Virginia Tech Carilion School of Medicine
Brigham and Women’s Hospital	Central Iowa Health System (Iowa Methodist Medical Center)	Exempla St. Joseph Hospital	College of Medicine Mayo Clinic (Jacksonville)
Brown University	Cleveland Clinic Foundation	Kaweah Delta Health Care District	Emory University
Dartmouth–Hitchcock Medical Center	College of Medicine Mayo Clinic (Rochester)	Loma Linda University	Greenville Hospital System/University of South Carolina School of Medicine
Georgetown University	Grand Rapids Medical Education Partners/Michigan State University	Los Angeles County-Harbor-UCLA Medical Center	Inova Fairfax Hospital/Inova Fairfax Hospital for Children
Hospital of St. Raphael	Gundersen Lutheran Medical Foundation	Naval Medical Center	Keesler Medical Center
Johns Hopkins University	Indiana University School of Medicine	Oregon Health and Science University	Medical University of South Carolina
Lehigh Valley Hospital Network/Pennsylvania State University	Loyola University Chicago	St. Joseph’s Hospital and Medical Center	Pitt County Memorial Hospital/East Carolina University Program/Vidant Medical Center/East Carolina University Program
Maine Medical Center	McGaw Medical Center of Northwestern University	Stanford University	Tulane University
Massachusetts General Hospital	Mercy Hospital Center	Swedish Medical Center/First Hill	University of Florida
Mercy Catholic Medical Center	Mount Carmel Health System	UCLA Medical Center	University of Mississippi Medical Center
Mount Sinai School of Medicine	Providence Hospital and Medical Center	University of California (Davis) Health System	University of North Carolina Hospitals
New York Presbyterian Hospital (Columbia Campus)	Rush University Medical Center	University of California (Irvine)	University of Oklahoma Health Sciences Center
New York Presbyterian Hospital (Cornell Campus)	St. Joseph Hospital	University of California (San Francisco)/Fresno	University of Tennessee College of Medicine at Chattanooga
Penn State University/Milton S Hershey Medical Center	St. Vincent Hospitals and Health Care Center	University of Colorado Denver	University of Tennessee Medical Center at Knoxville
Pinnacle Health Hospitals	TriHealth Good Samaritan Hospital	University of Washington	University of Texas Health Science Center (San Antonio)
Sinai Hospital of Baltimore	University Hospital/University of Cincinnati College of Medicine		University of Virginia
St. Barnabas Medical Center	University of Chicago		Vanderbilt University
St. Luke’s Hospital	University of Illinois College of Medicine at Chicago (Mount Sinai)		Virginia Commonwealth University Health System
SUNY Health Science Center at Brooklyn	University of Nebraska Medical Center College of Medicine		
Temple University Hospital	University of North Dakota		
UMDNJ New Jersey Medical School	University of Wisconsin		
UMDNJ Robert Wood Johnson Medical School	Wright State University		
University of Massachusetts			
University of Pennsylvania			
University of Rochester			
Waterbury Hospital Health Center			
York Hospital

**Fig. 2:**
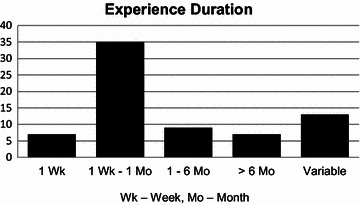
A *bar graph* demonstrating the typical lengths of international experiences. A majority of the available experiences are 1 week–1 month in length

**Fig. 3:**
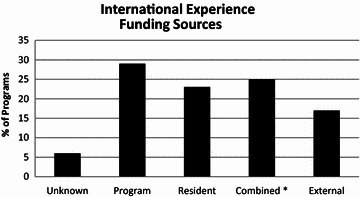
A *bar graph* demonstrating the funding sources for international experiences in the US. Resident, program or hospital, and combined funding each representing about a quarter of the funding sources with external funds and unknown sources accounting for the remaining quarter. *Asterisk* indicates combined funding included a combination of program, resident, and/or external funding

Further analysis revealed that most international experiences take place during the PGY 3 and PGY 4 level of training and last approximately 1–4 weeks (Fig. 2). Resident participation in international experiences is variable across programs ranging from 0 to >5 residents having participated to date (Table 4). Funding for these experiences is usually provided by the program, the resident, or a combination of funds (Fig. 3). These types of experiences have been available for >5 years in nearly half of the survey respondents. Most programs had 1 (n = 14), 2–4 (n = 16), or 5 (n = 12) residents who had participated in the international experience so far.

**Table 4:** Resident participation to date

Residents	Programs
Unknown	4
0	3
1	13
2–4	19
≥5	23

## Discussion

By the year 2030, it is estimated that surgical conditions will represent a substantial amount of the global disease burden. This can be attributed to the projected increase in cancer, road traffic injuries, cardiovascular, and metabolic disease (Mathers and Loncar 2006). Deaths due to road traffic injuries alone are expected rise to the 8th leading cause of mortality in low income settings. In addition, the Lancet Commission on Global Surgery estimates that 5 billion people worldwide do not have adequate access to surgical care. Furthermore, 143 million surgical procedures are needed in low and middle income countries (LMICs) to prevent disability and save lives (Meara and Greenberg 2015). In LMICs, nine out of ten people cannot access surgical care and, if they do reach care, their operative mortality is often significantly higher (Meara and Greenberg 2015; Crash Trial Collaborators et al. 2008). Without addressing this discrepancy, the gap between the delivery of care in low and middle income settings will continue to increase. One possible method to address this enhancing discrepancy is to increase and promote the use of twinning programs, international electives, and partnerships between high income countries and LMICs (Binagwaho et al. 2013; Olapade-Olaopa et al. 2014).

There is currently no study that describes in detail the international experiences available for general surgery residents across the US although previous studies have shown increasing resident interest in global health (Powell et al. 2007, 2009; Jayaraman et al. 2009). One recent study did review the availability of international experiences and the ease of finding this information on the websites of US general surgery residency programs. The study by Wackerbarth et al. (2015) reviewed the websites of 239 general surgery programs for an arbitrarily set time limit of 10 min. They noted that only a small number of programs (24 = 10 %) mentioned international experiences and a minority of programs (42 = 18 %) contained information about global surgery.

There are several differences between the study by Wackerbarth et al. (2015) and our current study. Although their study covered the majority of the general surgery residency programs, the information was obtained only through perusal of the websites for a limited period of time (10 min) which may not always be accurate or updated. Whereas, in our current study, we not only perused the websites without any time limit, but we also contacted each program individually to confirm the information regarding international experiences via e-mail or telephone calls. To enhance the accuracy of the information, individual programs were contacted directly on two occasions via e-mail or telephone at two separate time points. It is not surprising that the results of our study revealed that a higher number of programs (34 %) offered international experiences when compared to the study by Wackerbarth et al. In addition, the study by Wackerbarth et al. focused more on the presence of information on the websites and the ease of obtaining it rather than quantifying and characterizing the details of the international experiences. Their study did not address many other factors of importance relating to international experiences such as: geographic location of international experiences, source of funding, duration of existence of international experiences, PGY of international experience participation, and length of experiences. In contrast the main aim of our study is to identify, quantify, and characterize the details of the international experiences available.

We hope the results of the current study will fill this gap and provide useful information to general surgery residency programs and applicants. According to our results, the prevalence of international experiences in US general surgery residency training programs ranges from approximately 27–44 % across the four main National Resident Matching Program regions. Most of these experiences take place during the PGY 3 or PGY 4 levels of training and last approximately 1–4 weeks. The financing for these international experiences is fairly evenly divided between programs, residents, or a combined funding. A majority of these experiences occur in the Americas and Africa while experiences in Asia, Europe, and Australia are far less common. Our results on length and location of international experiences are consistent with those found by Mitchell et al. (2011) who found that international electives within US general surgery residency training programs averaged a length of 4 weeks and were most commonly located in Latin America.

As interest in international electives increases, it is also important to understand the educational benefits to the participating residents and participating institutions. International experiences complement the academic missions of service, training, and research (Meara and Greenberg 2015). They offer exposure to advanced pathology while teaching residents how to utilize sparse resources efficiently and confer an increased understanding of cultural differences affecting health care delivery (Ozgediz et al. 2005; Oliphant et al. 2012; Jarman et al. 2009). Additionally, in the age of increasing work hour regulation, international work has the potential to maximize the acquisition and improvement of technical and clinical skills over a relatively short period of time (Shrime et al. 2015). Experience abroad also opens up new avenues for international surgical research addressing epidemiology, natural history, and cost-effective treatment of surgical conditions in LMICs (Meara and Greenberg 2015). While a majority of the literature focuses on benefits to resident education, hosting institutions also receive benefit from international exchanges. O’Donnell et al. (2014) found that hosting institutions for international residents found improvement in knowledge sharing, transfer of medical knowledge, and formation of long-term relationships (Lukolyo et al. 2015). These benefits were most pronounced when the residents were toward the end of their training and focusing on their future practice and research goals. These established relationships also build partnerships for collaborative research with the goal of improving international medical care. Additionally, trainees were found to positively affect patient care, staff education, and promoting professionalism at host institutions. Despite the mutual benefits, trainees do pose some challenges to host institutions including decreased efficiency and negative perceptions of hosting countries held by trainees. These and other challenges can be minimized by preparing residents prior to arrival to a host institution with education on medical, ethical, and cultural challenges that may be encountered during international experiences (O’Donnell et al. 2014; Kraeker and Chandler 2013; Howe et al. 2013). Overall, these collaborations and partnerships are gateways to facilitate cross-cultural information exchange and enhance worldwide quality of care (Shrime et al. 2015; Klaristenfeld et al. 2008).

Although multiple educational benefits accompany international experiences, there are a number of barriers to programs wishing to establish such electives. These include establishing the initial contact with a foreign program, obtaining sources of funding, planning time for an international experience, and determining the ideal curriculum and length of the experience (Lett 2003). A rotation length of at least 4 weeks was seen as more beneficial than a shorter rotation by some host institutions (O’Donnell et al. 2014). Didactic training prior to international experiences also enhanced the experience for both trainee and hosting institution. Additionally, institutions must develop ways to continue adequate residency coverage while residents are participating in international electives (Klaristenfeld et al. 2008). Regardless of these obstacles, 78 % of program directors wanted information on establishing a partnership with a program abroad in a recent survey (Meara and Greenberg 2015). In that same study, 76 % of program directors were interested in information on funding models. This enthusiasm is encouraging for the further development of international resident activities.

Despite the commonly faced barriers, over a third of US general surgery residency programs have established formal or informal international electives. Our study is one of the first to document this information and has the best response rate from survey participants (94 %). It is worth noting that our study is not without limitations. Program directors and assistants were the main survey participants. A number of these participants stated that they were new to their institution and may not have complete knowledge of the international experiences available to residents in their programs. In addition, there may have been confusion on what qualifies as an international experience. Respondents may not have known about available informal international experiences including week-long experiences or mission trips led by faculty members. In these instances, they may have only answered yes if an official international rotation was available. Mitchell et al. (2011) classify formal rotations as “institutionally sponsored electives offered to all residents at some point during their training and considered to be part of the regular clinical elective schedule”. They found that 60 % of their 63 program director responses were for “informal” international experiences. Therefore, it is possible that this study underestimates the number of informal opportunities available to US general surgery residents.

## Conclusions

In conclusion, this is one of the first studies to attempt to identify, quantify, and characterize international experiences available to general surgery residents within the general surgery residency training programs in the US. In a time of growing demand for surgical care worldwide and an increasing global health interest among US residents, these results will help pave the way for the development of future collaborations between general surgery residency programs and residents interested in providing surgical care abroad. Ultimately, these electives will help to develop well-rounded and globally diverse surgeons who are better able to care for patients of all nationalities both locally and abroad.

## Abbreviations

US: United States
FREIDA: Fellowship and Residency Electronic Interactive Database
PGY: post graduate year
LMIC: low and middle income countries
